# Seasonal Characteristics and Source Apportionment of Water-Soluble Inorganic Ions of PM_2.5_ in a County-Level City of Jing–Jin–Ji Region

**DOI:** 10.3390/toxics14010017

**Published:** 2025-12-24

**Authors:** Shuangyun Guo, Lihong Ren, Yuanguan Gao, Xiaoyang Yang, Gang Li, Shuang Gao, Qingxia Ma, Yi Shen, Yisheng Xu

**Affiliations:** 1 State Key Laboratory of Environmental Criteria and Risk Assessment, Chinese Research Academy of Environmental Sciences, Beijing 100012, China; 2 Institute of Atmospheric Environment, Chinese Academy of Environmental Sciences, Beijing 100012, China; 3 Appraisal Center for Environment and Engineering, Ministry of Ecology and Environment, Beijing 100012, China; 4 Key Laboratory of Geospatial Technology for the Middle and Lower Yellow River Regions, Ministry of Education, College of Geography and Environmental Science, Henan University, Kaifeng 475004, China; 5 School of Earth Sciences, Yunnan University, Kunming 650500, China

**Keywords:** PM_2.5_, water-soluble inorganic ions, seasonal variation, source analysis, county-level city

## Abstract

Water-soluble inorganic ions (WSIIs) are major components of PM_2.5_ and play a prominent role in atmospheric acidification. Previous studies have mainly focused on urban areas, whereas research pertaining to county-level cities remains comparatively limited. To fill this gap, PM_2.5_ samples were collected from March 2018 to February 2019 in Botou, a county-level city in the Jing–Jin–Ji region. Seasonal variation of WSII were studied, and their sources was apportioned by Positive Matrix Factorization (PMF) model. Annual PM_2.5_ concentrations were 79.15 ± 48.44 mg/m^3^, which is 2.26 times of the Level II standard limit specified the National Ambient Air Quality Standard. Nitrate (NO_3_^−^) was the most abundant ion, followed by ammonium (NH_4_^+^) and sulfate (SO_4_^2−^). The secondary inorganic aerosols (SIA, i.e., SO_4_^2−^, NO_3_^−^, and NH_4_^+^) constituted 35.1± 4.7% of PM_2.5_ mass. PM_2.5_ mass, SO_4_^2−^, NO_3_^−^, NH_4_^+^, K^+^, and Cl^−^ showed highest concentrations in winter. Ammonium salts were existed as ammonium sulfate ((NH_4_)_2_SO_4_) and ammonium nitrate (NH_4_NO_3_) in spring, summer, and autumn, while it also can be existed as ammonium chloride (NH_4_Cl) in winter. PMF analysis shows that the sources of WSIIs dominated by secondary source and followed by biomass burning. These results highlight the need for improved controls on gaseous precursors (NH_3_, NO_2_ and SO_2_) and biomass burning to effectively reduce PM_2.5_.

## 1. Introduction

In recent years, with the implementation of pollution prevention and control measures, China’s air quality has been improved significantly, and the concentration of PM_2.5_ has dropped significantly. However, PM_2.5_ in many cities has not yet attained Class 2 standard of the *Ambient Air Quality Standards*. According to the *2022 Bulletin on the State of the Ecology and Environment in China* [1], 25% of Chinese cities have PM_2.5_ levels that exceed the established standard, and there is a long way to go to control PM_2.5_ pollution. Water-soluble inorganic ions (WSIIs) are an important part of atmospheric PM_2.5_, accounting for more than 20–70% of PM_2.5_ mass concentration. WSIIs can affect the acidity of atmospheric precipitation [2], reduce atmospheric visibility [3], affect the optical properties of aerosols, and impact the earth’s radiation balance by absorbing and diffusing solar radiation [4]. Therefore, the research on chemical characteristics of water-soluble inorganic ions is helpful for understanding their chemical characteristics and behaviors, as well as the formation mechanism of environmental aerosols.

The composition of PM_2.5_ is notably complex, including a variety of trace elements, water-soluble inorganic ions, organic carbon, inorganic carbon, and other substances. Among these components, WSIIs are an important component of PM_2.5_, accounting for one-third or more of PM_2.5_ [5,6,7]. Currently, extensive research has been undertaken to explore the chemical properties, formation mechanisms, and origins of WSIIs in atmospheric PM_2.5_. However, variations in geographical conditions, energy structures, meteorological factors, resident’s lifestyles, and industrial structures across different regions result in diverse formation mechanisms of PM_2.5_ pollution. This diversity, in turn, leads to significant differences in the chemical composition and concentration levels of WSIIs between urban and background regions [8]. Furthermore, previous studies have mainly focused on urban areas [9,10,11,12], whereas research pertaining to county-level cities remains comparatively limited.

In recent years, with adjustments to the industrial structure of large- and medium-sized cities in China, the transfer of traditional industries to county-level regions, and the growing agglomeration of population in county seats, the focus of urbanization has shifted from cities to surrounding county-level cities. Notably, as a direct consequence, the air pollution issue in county-level cities has garnered increasing scholarly and regulatory attention. Since county-level cities have a greater diversity of potential pollution sources than urban areas and their environmental regulatory capacity is lower than that of urban areas, air pollution in county-level cities tends to be more pronounced. Although the PM_2.5_ concentration in most urban areas in China has shown a significant downward trend, the annual average PM_2.5_ mass concentration in 2640 county-level cities in China increased from 29.52 mg/m^3^ to 42.83 mg/m^3^ between 2000 and 2010 [13]. Notably, this upward trend has persisted through subsequent years. By 2020, the total resident population in China’s county-level cities had reached 745 million [14], reflecting their significant demographic weight. Therefore, there is an urgent need to investigate the chemical characteristics of PM_2.5_ water-soluble inorganic ions, with a specific focus on county-level cities.

As a core political, economic, and developmental hub, the Jing-Jin-Ji region has experienced a substantial surge in energy consumption amid sustained socioeconomic development. This change has exerted adverse impacts and severe pressure on the environment of this region. The average annual PM_2.5_ mass concentration in the Jing-Jin-Ji region was 64.9 mg/m^3^ in 2017 [15]. At present, research on PM_2.5_ in the Jing-Jin-Ji region mainly focuses on urban areas, such as Beijing [16], Tianjin [17,18], Shijiazhuang [19,20], and Tangshan [20], while studies targeting county-level cities are relatively scarce. In this study, ambient PM_2.5_ samples were collected in Botou (a county-level city administered by Cangzhou, in Jing-Jin-Ji region, which lies in proximity to Beijing and Tianjin and borders the Bohai Sea to the east) between 2018 and 2019 covering a one-year period to capture seasonal variability. The research was designed to investigate the seasonal variability of PM_2.5_-associated water-soluble ions and the formation mechanisms of secondary aerosols in Botou. Furthermore, the Positive Matrix Factorization (PMF) model was employed to quantify and apportion the key sources of PM_2.5_-associated water-soluble ions. The results are expected to provide empirical data and a theoretical foundation for formulating targeted PM_2.5_ abatement strategies in Botou and even the Jing-Jin-Ji region. Moreover, the results offer valuable insights for air quality management in other county-level cities with similar industrial and geographic contexts.

## 2. Materials and Methods

### 2.1. Research Area and Sample Collection

**Figure 1:**
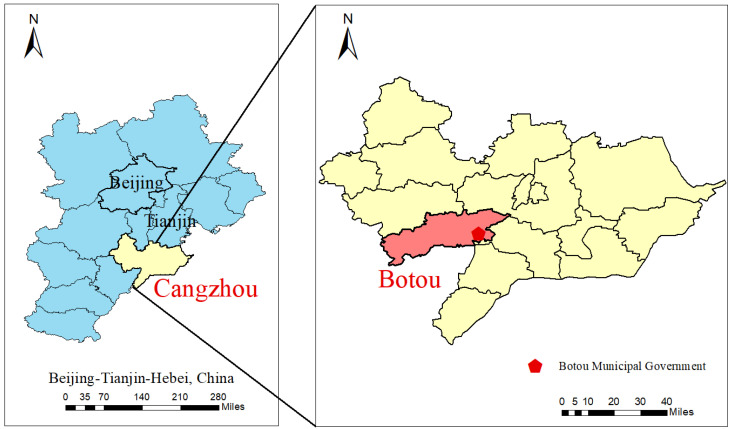
Distribution map of sampling point.

The sampling site is situated on the rooftop of the Botou Municipal Government (38.084° N; 116.578° E), with close proximity to a provincial-level ambient air quality monitoring station, as shown in Figure 1. The site is approximately 10 m above ground level and is surrounded by residential districts and major urban roadways, endowing it with strong spatial representativeness for ambient air quality. In this study, a TH-16A four-channel intelligent ambient air particulate matter sampler (Wuhan Tianhong Instrument Co., Ltd, Wuhan, China) was used for ambient PM_2.5_ samples collection, with a sampling flow rate of 16.7 L·min^−1^. Two of the four channels were equipped with 47 mm diameter PTFE (polytetrafluoroethylene) and quartz fiber filters respectively for sampling in parallel. PTFE filter was primarily used for mass determination and inorganic element quantification, while quartz filters were primarily used for carbonaceous component analysis and the target water-soluble ion analysis. For the purposes of this study, only the characteristics of water-soluble ions were characterized. To ensure the representativeness and temporal continuity of the dataset, the sampling period was from March 2018 to February 2019, encompassing four distinct seasons, with daily sampling conducted from 10:00 to 09:00 the following day (23 h sampling duration). A total of 202 valid samples were obtained, after excluding samples affected by rainfall, snow, power outages, or equipment maintenance.

### 2.2. Sample Analysis

A 1/8 portion was cut from the quartz fiber filter, immersed in 20.00 mL of 18.2 MΩ.cm deionized water, vortexed thoroughly, and then placed in an ultrasonic bath (40 KHz frequency, 25 ± 2 °C) for 15 min of extraction. Following a 5 min equilibration period, the supernatant was collected via vacuum filtration through a 0.22 µm nylon syringe filter for subsequent ion chromatography (IC) analysis. In this study, a ion chromatography (ICS-900, Dionex Corporation, Sunnyvale, MA, USA) was used for the quantitative determination of 9 major target water-soluble ions in the samples, including Na^+^, Mg^2+^, Ca^2+^, K^+^, NH_4_^+^, SO_4_^2−^, F^−^, Cl^−^, and NO_3_^−^. The separation column utilized was a Dionex IonPac^TM^ AS22 analytical column (Dionex Corporation) (4 × 250 mm), equipped with a Dionex IonPac^TM^ AG22 guard column (Dionex Corporation) (4 × 50 mm); the eluent was a mixed solution of 4.5 mM Na_2_CO_3_ and 1.4 mM NaHCO_3_, delivered at a flow rate of 1.0 mL × min at 30 °C. Ultrasonic-assisted extraction was selected to enhance the leaching efficiency of water-soluble ions from the solid filter matrix, which is a well-established pretreatment approach for IC analysis of atmospheric particulate matter. Stringent quality assurance (QA) and quality control (QC) measures were implemented throughout the analytical process to ensure data reliability, in compliance with environmental protection standards of China [21,22]. These measures included: (1) procedural blanks (*n* = 10) analyzed alongside samples to correct for background contamination; (2) duplicate samples (10% of total samples) with relative standard deviations (RSDs) < 5%; (3) calibration curves for each ion with correlation coefficients (R^2^) ≥ 0.999; and (4) spiked recovery tests ranging from 85% to 115%.

### 2.3. Sources of Gaseous Pollutants and Meteorological Data

Conventional pollutant data (SO_2_, NO_2_, O_3_) used in the study were all obtained from the automatic monitoring datasets from nearby national ambient air quality monitoring stations (NAQMS) in proximity to the filter sampling site. Key meteorological data (relative humidity, temperature) were acquired from the Cangzhou Meteorological Bureau with an hourly time resolution to match the temporal coverage of sampling and pollutant monitoring.

### 2.4. Sulfur Oxidation Ratio (SOR) and Nitrogen Oxidation Ratio (NOR)

The SOR and NOR are widely used to quantify the conversion degree of gaseous SO_2_ and NO_X_ to particulate SO_4_^2−^ and NO_3_^−^ in atmospheric particles [23]. When SOR or NOR exceed 0.1, the SO_4_^2−^ and NO_3_^−^ in atmospheric PM_2.5_ predominantly originate from the gas-to-particle secondary conversion of SO_2_ and NO_2_ [23]. The higher the SOR or NOR value, the greater the conversion degree of gaseous SO_2_ and NO_2_ to form secondary aerosol components. Their respective calculation equations are defined as follows:

(1) $$\begin{array}{c} SOR=\frac{c\left({SO}_{4}^{2-}\right)}{c\left({SO}_{4}^{2-}\right)+c\left({SO}_{2}\right)} \end{array}$$

(2) $$\begin{array}{c} NOR=\frac{c\left({NO}_{3}^{-}\right)}{c\left({NO}_{3}^{-}\right)+c\left({NO}_{2}\right)} \end{array}$$

where, $c({SO}_{4}^{2-})$ and $c({NO}_{3}^{-}$) denote the mass concentrations of particulate sulfate and nitrate ions in the atmosphere. Meanwhile, c(SO_2_) and c(NO_2_) represent the mass concentrations of SO_2_ and NO_2_. The unit of $c\left({SO}_{4}^{2-}\right),c({NO}_{3}^{-}$), c(SO_2_) and c(NO_2_) was μmol/m^3^.

### 2.5. Positive Matrix Factorization (PMF)

PMF is a widely used receptor-oriented approach for particulate matter source apportionment, derived from traditional factor analysis [24]. It leverages ambient observational dataset from receptor sites to quantify the chemical profiles of potential pollution sources and their respective contributions to total ambient pollutant concentrations. First, the PMF model quantifies the uncertainty associated with each individual chemical component in particulate matter based on component-specific weights, and then identifies the dominant pollution source categories and quantifies their contribution ratios via an iterative least squares algorithm. Its core principle involves factorizing the original concentration matrix ${\;X}_{nm}$ into two factor matrices, specifically the source contribution matrix $G_{np}$ and the source profile matrix $F_{pm}$, along with a residual matrix $E_{nm}$ that accounts for discrepancies between observed and modeled concentrations. The mathematical expression is defined as follows:

(3) $$\begin{array}{c} X_{n\times m}=\sum_{k=1}^{p}\;G_{n\times p}F_{p\times m}+E \end{array}$$

In the formula, $X_{n\times m}$ represents the concentration of the *m*-th chemical component in the *n*-th sample (µg/m^3^), while p denotes the number of identified pollution source categories (factors) derived from the analysis. Matrices *G_n×p_* and *F_p×m_* correspond to the source contribution matrix and the source profile matrix, respectively, with all elements restricted to non-negative values—a key constraint of the PMF algorithm that aligns with the physical meaning of source contributions and chemical profiles.

Following iterative runs of the PMF model, the optimal *Q* value (representing the goodness-of-fit of the model) is derived, thereby confirming the final source profile matrix (*F*) and the source contribution matrix (G). The mathematical expression for the Q function is defined as follows:

(4) $$\begin{array}{c} Q=\sum_{i=1}^{m}\;\sum_{j=1}^{n}\;{\left(\frac{E_{ij}}{\sigma_{ij}}\right)}^{2} \end{array}$$

where $\sigma_{ij}$ denotes the analytical uncertainty (or standard deviation) of the *j*-th chemical component in the *i*-th sample, and $E_{ij}$ is the residual term between the observed and modeled concentration of the *j*-th component in the *i*-th sample data.

## 3. Results and Discussion

### 3.1. Seasonal Variation of PM_2.5_ Mass Concentration

**Figure 2:**
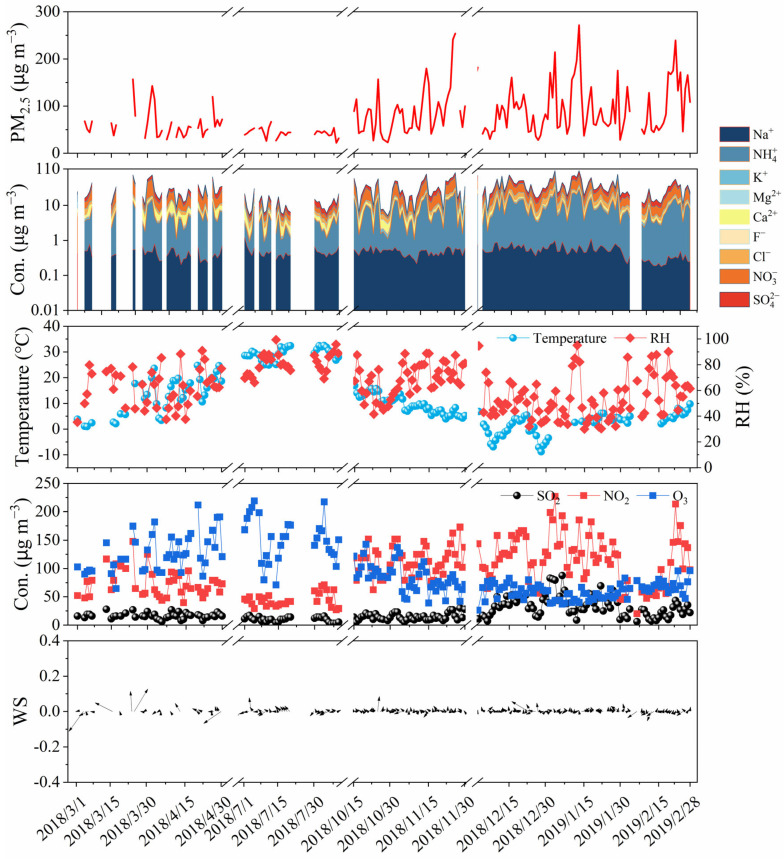
Time series of PM_2.5_ mass concentration, WSIIs, gaseous pollutants, and meteorological paremeters at sampling site.

Figure 2 present the time variations of PM_2.5_ mass concentration, WSIIs, target gaseous pollutants, and key meteorological parameters during the sampling period. Throughout the entire observation period, the annual average value of PM_2.5_ in Botou was (79 ± 48) μg/m^3^, which is 2.26 times the annual Level II standard limit for PM_2.5_ (35 μg/m^3^) specified the National Ambient Air Quality Standard (GB 3095-2012) [25] of China and higher than the corresponding annual mean PM_2.5_ concentration in Beijing during the same 2018–2029 period (67 ± 60 μg/m^3^) [26].

The PM_2.5_ concentration exhibits distinct seasonal variability, with the descending order: winter (95 ± 53 μg/m^3^) > autumn (87 ± 50 μg/m^3^) > spring (63 ± 32 μg/m^3^) > summer (43 ± 9 μg/m^3^). This seasonal pattern is strongly associated with seasonal variation in meteorological conditions and anthropogenic emission. The notably elevated PM_2.5_ concentration in winter is mainly attributed to enhanced anthropogenic emissions driven by centralized heating. Furthermore, unfavorable atmospheric diffusion conditions promote the accumulation and transport of primary pollutants and secondary transformation of gaseous precursors [27]. Notably, the maximum daily PM_2.5_ concentration recorded on January 3, 2019, reached 193.42 μg/m^3^. The meteorological conditions on that day were characterized by an ambient relative humidity of 59% and calm wind conditions (wind speed 0.82 m/s), which further inhibited pollutant diffusion. Spring PM_2.5_ is also moderately elevated, mainly affected by regional dust events (e.g., sandstorms, blowing dust, and floating dust). In summer, enhanced wind speeds and increased precipitation frequency favor pollutant diffusion and wet deposition, thereby effectively lowering ambient PM_2.5_. The seasonal variation characteristics of PM_2.5_ in Botou are consistent with that of major cities in Jing-jin-ji region such as Shijiazhuang [28], reflecting the regional consistency of PM_2.5_ pollution drivers in Jing-jin-ji region.

### 3.2. Seasonal Variation of WSIIs

Figure 2 and Table 1 present the annual average concentration and seasonal variation characteristics of WSIIs. During the observation period, the annual average concentration of the total water-soluble inorganic ions in Botou was (29.67 ± 19.39) μg/m^3^, accounting for 44.43% of the ambient PM_2.5_ mass concentration, indicating a substantial contribution of WSIIs to PM_2.5_ mass. The individual WSIIs follow this order, in descending order of concentration: NO_3_^−^ > NH_4_^+^ > SO_4_^2−^ > Cl^−^ > Ca^2+^ > K^+^ > Na^+^ > F^−^ > Mg^2+^. The annual average concentration of SIA was 24.51 μg/m^3^, accounting for about 35% of the total PM_2.5_ mass concentration, which are the primary components of secondary aerosols, indicating their dominant contribution to PM_2.5_. Specifically, NO_3_^−^, SO_4_^2−^, and NH_4_^+^ accounted for 15%, 10%, and 10% of the PM_2.5_ mass concentration, respectively. Compared with SIA, the contribution ratios of Cl^−^, Ca^2+^ and K^+^ are relatively minor, with contribution ratios of 4%, 2%, and 1% respectively, while the proportions of Na^+^, F^−^, and Mg^2+^ are all less than 1%.

**Figure 3:**
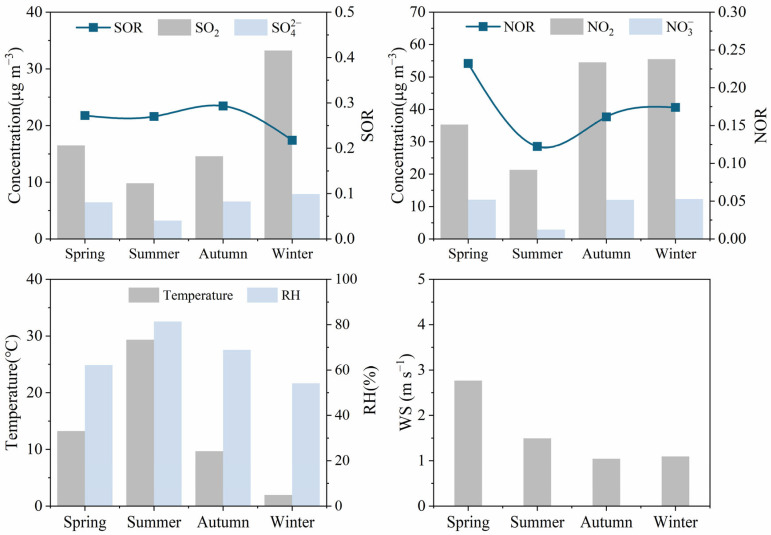
Seasonal distribution of SOR and NOR in Botou.

NO_3_^−^ showed the highest concentration in spring, autumn, and winter (12.07–12.32 μg/m^3^) and the lowest in summer (2.87 μg/m^3^). It is noted that the concentration variations of NO_3_^-^ were much higher than its precursor NO_2_. The maximum concentration of NO_3_^-^ was observed in autumn and winter, which was 4.31–4.21 times higher than summer, while the concentration of its precursor NO_2_ was 2.56–2.60 times higher in autumn and winter than in summer. The seasonal variation of NO_3_^-^ concentration reflects the seasonal changes in its precursor NO_2_ and the seasonal impact on thermodynamic equilibrium. In summer, NO_X_ is prone to form HNO_3_ through photochemical reactions, which further leads to the formation of secondary particles, as shown in Formula 5. But elevated ambient temperature enhances the thermal dissociation of ammonium nitrate (NH_4_NO_3_), and thus reduces the concentration of particulate NO_3_^−^ [2]. Although the photochemical activity of the atmosphere is not strong in autumn and winter, high NO_2_ concentration, the relatively low temperature and certain relative humidity are conducive to the secondary formation of NO_3_^−^. Gaseous HNO_3_ tends to react with NH_3_ for neutralization, and then convert into NH_4_NO_3_ under low-temperature conditions. According to the findings of Stockwell et al. [29], a study published in 2000 showed that approximately 33% (mole fraction) of the NO_X_ emitted during winter in the San Joaquin Canyon region of central California, USA, was converted into particulate matter. Furthermore, the elevated PM_2.5_ concentrations in these two seasons provide increased aerosol surfaces and elevated concentrations of the gaseous precursor (NO_2_) concentration for nitrate heterogeneous formation reactions, thereby promoting the heterogeneous secondary formation of NO_3_^−^ [30]. The comparable concentration of NO_3_^−^ in spring, relative to autumn and winter, was due to a higher NOR, even though the NO_2_ concentration was lower (Figure 3).

(5) $${NO}_{2}+OH+M\to {HNO}_{3}+M$$

The seasonal variation of SO_4_^2−^ exhibits a variation pattern in winter (7.89 μg/m^3^)> autumn (6.59 μg/m^3^) > spring (6.46 μg/m^3^) > summer (3.24 μg/m^3^) (Table 1 and Figure 3). The SO_4_^2−^ concentrations in winter were 2.43 times of that in summer. The highest SO_4_^2-^ concentration in winter is related to enhanced emission of gaseous precursor (SO_2_) from residential coal combustion for winter heating. Although photochemical transformation is limited in this season, substantial SO_2_ can still be oxidized to generate significant amounts of SO_4_^2−^ via reactions in cloud/fog droplets under conditions of low temperature and certain humidity [31], as shown in Formulas 6 and 7. It has been demonstrated that elevated levels of humidity can expedite the transformation of SO_2_ into particulate matter, thereby amplifying the formation of SO_4_^2–^. While summer exhibits higher temperatures, stronger photochemical oxidation capacity, and a higher SOR compared to other seasons, the concentration of SO_4_^2−^ remains lower than in other seasons. This is primarily attributable to the lower SO_2_ concentration in summer, further underscoring the significant influence of local SO_2_ on emissions on sulfate formation. The seasonal variation trend of SO_4_^2−^ in Botou differs from that in Beijing [32], where SO_4_^2−^ concentration peaks in summer and reaches its lowest in winter, largely due to the stronger SOR in summer.

(6) $${SO}_{2}+H_{2}O↔H^{+}+{HSO}_{3}^{-}$$

(7) $${HSO}_{3}^{-}+H_{2}O_{2}\to {SO}_{4}^{2-}+H^{+}+H_{2}O$$

The seasonal variation of NH_4_^+^ is similar to SO_4_^2−^: winter (9.33 μg/m^3^) > autumn (7.36 μg/m^3^) > spring (5.08 μg/m^3^) > summer (2.54 μg/m^3^). NH_4_^+^ strongly linked to the secondary formation of the ionic species SO_4_^2−^ and NO_3_^−^, primarily through the neutralization of acidic precursors to form ammonium salts (e.g., (NH_4_)_2_SO_4_, NH_4_NO_3_).

Cl^−^ and K^+^ exhibits similar seasonal variation pattern with highest concentration in winter (3.63 μg/m^3^ for Cl^−^ and 1.05 μg/m^3^ for K^+^) and the lowest in summer (1.16 μg/m^3^ for Cl^−^ and 0.27 μg/m^3^ for K^+^). Their concentration in spring and autumn was close. As a land-based city, the main source of Cl^−^ in Botou is coal combustion [33]. K^+^ mainly comes from biomass combustion [34]. The highest concentration of Cl^−^ and K^+^ in winter is associated with increased emissions during the heating period and stable meteorological conditions [35].

Ca^2+^ and Mg^2+^ reach their peak concentrations in spring (0.22 μg/m^3^ and 1.85 μg/m^3^), which may be related to the low precipitation, strong wind and sandstorms, or floating weather caused by the Mongolian monsoon in spring.

To further investigate the concentration levels of water-soluble ions in PM_2.5_ in Botou, we compare them with the concentration levels of WSIIs in PM_2.5_ from other cities in China, as shown in Table 2. The mass concentrations of PM_2.5_ and WSIIs in Botou are relatively lower than many prefecture-level cities, such as Hefei, Handan, Xi’an, Taiyuan, and Urumqi.

### 3.3. Existing Formation of SIA

**Figure 4:**
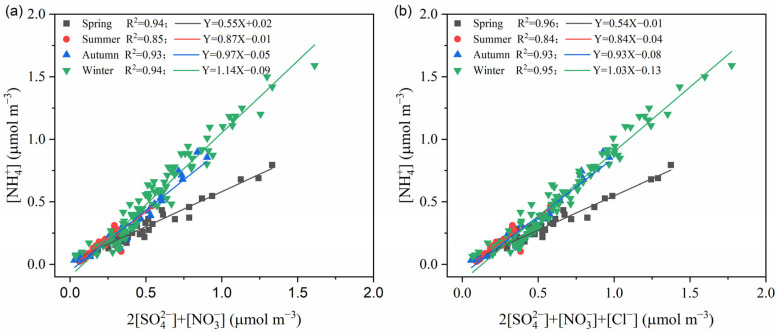
Scatter plots of [NH_4_^+^] V.S. (2[SO_4_^2−^] + [NO_3_^−^]) (**a**) and [NH₄₊] V.S. (2[SO₄²⁻] + [NO₃⁻] + [Cl^−^]) (**b**) in different seasons.

Research has demonstrated that NH_4_^+^ is the most important alkaline ion in PM_2.5_. The initial step in the process involves the combination of the acidic ion SO_4_^2−^ with (NH_4_)_2_SO_4_ and NH_4_HSO_4_. Thereafter, the remaining NH_4_^+^ reacts with NO_3_^−^ and Cl^−^ ions [39]. In all four seasons of Botou, the molar concentration ratios of NH_4_^+^ to SO_4_^2−^ are all greater than 2, being 2.10, 2.09, 2.98, and 3.15 respectively, indicating the PM_2.5_ was ammonia-rich state. This finding suggests that the presence of SO_4_^2−^ and NH_4_^+^ is indicative of a complete combination reaction, resulting in the formation of (NH_4_)_2_SO_4_. This process is accompanied by the retention of excess NH_4_^+^. To further analyze the existing forms of ammonium salts in Botou across the four seasons, we examine the correlations between [NH_4_^+^] and 2[SO_4_^2−^] + [NO_3_^−^] (Figure 4). In spring and summer, a robust correlation is observed between [NH_4_^+^] and the combined concentrations of [SO_4_^2−^] + [NO_3_^−^]. The slopes of the fitting curves were 0.55 and 0.87 respectively, suggesting that [SO_4_^2−^] and [NO_3_^−^] are in excess while the cation [NH_4_^+^] is insufficient in these two seasons. Thus, ammonium salts mainly exist in the forms of (NH_4_)_2_SO_4_ and NH_4_NO_3_ in these two seasons, and the remaining [SO_4_^2−^] and [NO_3_^−^] may react with other cations. In autumn, the slope between [NH_4_^+^] and 2[SO_4_^2−^] + [NO_3_^−^] is 0.97, which is close to 1, meaning ammonium salts primarily exist as (NH_4_)_2_SO_4_ and NH_4_NO_3_. During the winter months, the slope increases to 1.14, suggesting an excess of NH_4_^+^. Meanwhile, the slope of the fitting curve between [NH_4_^+^] and 2[SO_4_^2−^] + [NO_3_^−^] + [Cl^−^] is 1.03, which is proximate to 1. This suggests that ammonium salts in winter mainly exist in the forms of (NH_4_)_2_SO_4_, NH_4_NO_3_, and NH_4_Cl.

### 3.4. Analysis of WSIIs Sources

#### 3.4.1. NO_3_^−^/SO_4_^2−^ Ratio

SO_4_^2−^, a constituent of atmospheric composition, is predominantly derived from the secondary transformation of SO_2_ and emissions resulting from coal combustion [41]. NO_3_^−^ is principally derived from the secondary transformation of NO_2_ in exhaust gases emitted by vehicles and other means of transportation [32]. Therefore, the ratio of these two ions can be used to compare the contribution of mobile sources and stationary sources to nitrogen and sulfur in the atmosphere [42,43]. During the study period, the seasonal variation characteristics of the NO_3_^−^/SO_4_^2−^ ratio exhibited the following order of magnitude: spring (1.9) > autumn (1.7) > winter (1.5) > summer (0.9). The ratio of NO_3_^−^/SO_4_^2−^ during spring, autumn and winter is all greater than 1, indicating that the contribution of mobile sources is greater than that of stationary sources in these seasons. In contrast, the ratio in summer was less than 1, which may be attributable to the decomposition and volatilization of nitrate induced by the elevated temperatures characteristic of the summer season. This process results in a decline in the NO_3_^−^ concentration. Furthermore, elevated temperatures and humidity levels during the summer months promote the formation of SO_4_^2−^, thereby further diminishing the NO_3_^−^/SO_4_^2−^ ratio [44]. In this study, the concentration level of NO_3_^-^ was generally higher than that of SO_4_^2-^. This finding suggests that the regulatory measures implemented in Botou, including the oversight of coal-fired enterprises such as power plants and heating facilities, along with the management of dispersed coal combustion by residents in surrounding rural areas, have yielded initial outcomes. The issue of SO_2_ emissions have been effectively controlled. However, NO_X_ pollution, mainly from motor vehicle emissions, remains at a relatively high level [45].

#### 3.4.2. Ion Correlation Analysis

**Figure 5:**
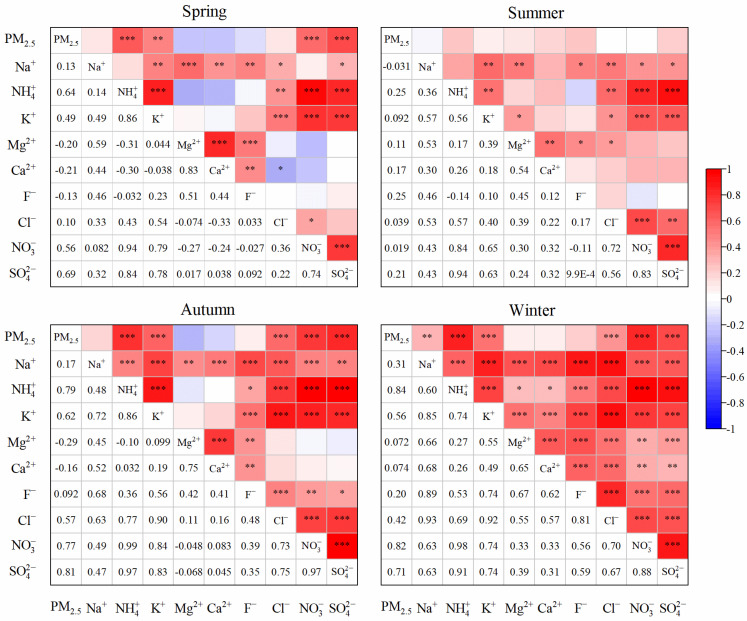
Spearman correlations of PM_2.5_ and water-soluble ions. Note: * indicates significance: * indicates *p* < 0.05; ** indicates *p* < 0.01; *** indicates *p* < 0.001.

In order to more thoroughly investigate the sources of WSIIs in PM_2.5_ of Botou and the correlation characteristics between each ion, this study conducts a correlation analysis on WSIIs. As shown in Figure 5, SIA exhibites significant correlations in all four seasons (correlation coefficients all greater than 0.8), indicating that the primary pollution sources and secondary transformation accumulation processes of the three ions are similar. In addition, SIA has been shown to exhibit a notable correlation with PM_2.5_ in autumn and winter, which suggests that secondary conversion has a significant impact on the formation of PM_2.5_ [46]. NH_4_^+^ demonstrates a substantial correlation with SO_4_^2−^, NO_3_^−^, and Cl^−^ throughout the year. This phenomenon may attributed to the propensity of NH_4_^+^ to form complexes with SO_4_^2−^ and NO_3_^−^. And the excess NH_4_^+^ combines with Cl^−^ to form NH_4_Cl, which is consistent with the previous analysis. Ca^2+^ is a tracer for construction and road dust [47]. A significant correlation has been observed between Ca^2+^ and Mg^2+^ in all four seasons, implying that these two ions may have a common origin, potentially derived from dust sources such as soil and construction activities. A moderate correlation between K^+^ and Cl^−^ was observed in summer, with a coefficient of determination of r = 0.4. This correlation was found to be significant in other seasons as well. This result suggests that these two water-soluble ions may have the same sources throughout the year, such as biomass burning sources, mineral dust, and fossil fuel combustion sources [48]. Throughout the year, Na^+^ and Cl^−^ exhibit a relationship characterized by the shared presence of a common source, which may be attributed to sea salt or sea fog. K^+^ and Ca^2+^ only show a certain correlation in winter with a relatively low correlation coefficient, and no obvious correlation is observed in the other three seasons. This indicates that although mineral dust contributes to the source of K^+^, it is not the main source of K^+^. There is no significant correlation in the other three seasons. Although mineral dust contributes to the source of K^+^, it is not its main source.

#### 3.4.3. PMF Analysis

**Figure 6:**
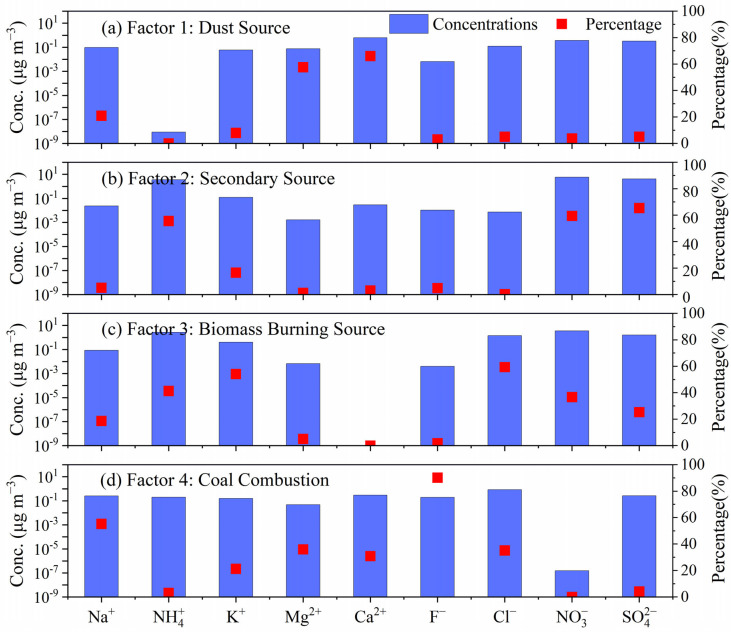
PM_2.5_ factor spectrum in Botou.

Based on the EPAPM5.0 software, this study conducts source apportionment of WSIIs during the observation period and identifies four main pollution source factors. As shown in Figure 6, the PMF model results indicate that the measured total mass concentration of WSIIs has a high correlation with the fitted total mass concentration of WSIIs (R^2^ = 0.98). The ratio of Q target value to Q theoretical value is 1.05, which proves that the source apportionment results obtained in this study are reasonable.

In factor 1, the contributions of Ca^2+^ (66%) and Mg^2+^ (58%) are significant. The presence of these two ions is typically attributed to construction, soil disturbance, and road dust emissions [49,50]. Therefore, factor 1 is identified as the dust source. In factor 2, SO_4_^2−^ (65%), NO_3_^−^ (59%), and NH_4_^+^ (56%) have relatively high contribution. These ions mainly come from the secondary conversion process of pollutants such as SO_2_ and NO_X_ [51,52]. Therefore, factor 2 is regarded as the secondary source. Factor 3 is characterized by the highest contributions of Cl^−^ (59%) and K^+^ (54%), while NO_3_^−^ (37%) and SO_4_^2−^ (25%) also have relatively large contributions. It is important to note that K^+^ is frequently utilized as a tracer ion in the study of biomass burning [53]. The KCl produced by biomass burning may react with NO_3_^-^ and SO_4_^2−^ form K_2_SO_4_ and KNO_3_ during atmospheric transport. Factor 3 is thus classified as the biomass burning source. Finally, factor 4 demonstrates the highest proportion of F^−^ (90%), while Na^+^ (55%), Cl^−^ (35%), and Mg^2+^ (36%) also account for a considerable proportion. It has been determined that F^−^ is mainly derived from waste incineration and coal combustion activities. Consequently, factor 4 is identified as the coal combustion source.

**Figure 7:**
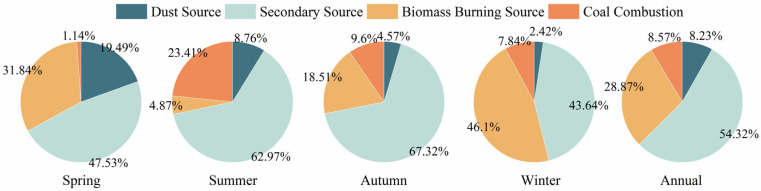
Sources apportionment of water-soluble ions in different seasons in Botou.

A notable seasonal variation is evident in the contribution of different pollution sources to PM_2.5_ in Botou, as shown in Figure 7. The secondary source accounts for a relatively high proportion in all seasons with the high proportion in autumn reaching 67.32%, while it drops to 43.64% in winter. This trend indicates that the secondary source makes a particularly significant contribution to PM_2.5_ in Botou highlighting that the control of precursor substances of secondary pollutants is the key to improving the air quality in Botou. The contribution of the biomass burning source is relatively prominent in spring and winter, accounting for 31.84% and 46.1%. The contribution exhibits a minimum in the summer months, with a contribution of 4.87%. This phenomenon may be associated with the substantial incineration of crop residue during the spring and winter seasons. This results in an augmented contribution from biomass burning sources during these periods. The contribution of coal combustion source is the highest in summer (accounting for 23.4%) and the lowest in spring (accounting for 1.14%). The predominant rationale for the increased proportion of coal combustion source during summer may is likely associated with the diminished contribution of biomass burning. The decline in straw burning during the summer months has led to an increase in the contribution of coal combustion as a source of air pollutants. In addition, the seasonal variation of dust source shows a relatively high proportion in spring, reaching 19.49%. This phenomenon may be attributed to the recurrent occurrence of sandstorms during the spring months, which leads to an augmented contribution of dust particles to the PM_2.5_ concentration.

To summarize, the atmospheric pollution in Botou exhibits a multifaceted pattern of contamination, with dust source contributing 8.2%, secondary source accounting for 54.3%, biomass burning source representing 28.9%, and coal combustion source representing 8.6%. A comprehensive review of the extant literature reveals that secondary source has been identified as the most significant contributor to fine particulate matter, a finding consistent with research results from Xi’an [6] and Lüliang [54]. This finding underscores the pivotal role of emission reduction and control measures for the gaseous precursors of secondary sources are the key to tackling Botou’s atmospheric pollution. In addition, biomass burning makes a significant contribution in winter and spring seasons. It is recommended to strengthen the management of crop straw burning during these two seasons to reduce the contribution of biomass burning to PM_2.5_. The implementation of targeted control strategies has been identified as a pivotal approach for effectively mitigating PM_2.5_ concentrations in Botou, thereby enhancing the city’s air quality.

## 4. Conclusions

The annual average mass concentration of PM_2.5_ is (79 ± 48) μg/m^3^ during the observation period. The PM_2.5_ demonstrate a marked seasonal variation, characterized by the following descending order: winter > autumn > spring > summer. The total concentration of WSIIs is highest in winter and lowest in summer, which is mainly associated with winter heating activities and unfavorable atmospheric diffusion conditions. The sum of SO_4_^2−^, NO_3_^−^, and NH_4_^+^ constitute 35± 4% of PM_2.5_ mass. The proportion of secondary aerosols is notably high, indicating their dominant contribution. This result underscores the necessity to prioritize the management of their precursor substances, including SO_2_, NO_2_, and NH_3_, in order to effectively address the issue of air quality. PM_2.5_ in Botou has been determined to be an ammonia-rich state on an annual average basis. The ammonium salts have been identified as (NH_4_)_2_SO_4_ and NH_4_NO_3_ in spring, summer, and autumn, while it also can exist as (NH_4_Cl) in winter. The NO_3_^−^/SO_4_^2−^ ratio indicates that the contribution of mobile sources in Botou is greater than that of stationary sources in spring, autumn, and winter. This shows that NO_X_ pollution remains at a relatively high level. Throughout the year, significant correlations have been observed among SIA components, with all components demonstrating notable associations with PM_2.5_. These findings suggest a predominant relationship between PM_2.5_ and the secondary generation of aerosols. PMF analysis shows that the air pollution in Botou is characterized by a mixed pollution pattern dominated by secondary source, biomass burning source, coal combustion source and dust source. Among them, secondary source is the main source, so emission reduction and control measures for gaseous precursors (NH_3_, NO_2_ and SO_2_) from secondary source are the key to controlling air pollution in this region. Subsequent research should integrate meteorological parameters (relative humidity, temperature), aerosol water content, and aerosol acidity to thoroughly investigate the formation mechanisms and influencing factors of SIA. Moreover, this study concentrated on water-soluble ions; henceforth, endeavors should incorporate carbon components and inorganic elements to systematically examine changes in the chemical characteristics of PM_2.5_.

## Figures and Tables

**Table 1:** Seasonal variation of water-soluble ion composition in PM_2.5_ (μg/m^3^).

Component	Annual	Spring	Summer	Autumn	Winter
Na^+^	0.48 ± 0.17	0.42 ± 0.14	0.47 ± 0.17	0.48 ± 0.11	0.51 ± 0.21
Cl^−^	2.51 ± 1.79	1.83 ± 0.92	1.16 ± 0.16	1.91 ± 0.84	3.63 ± 2.13
Ca^2+^	1.04 ± 0.82	1.85 ± 1.31	0.88 ± 0.63	1.00 ± 0.34	0.71 ± 0.35
Mg^2+^	0.14 ± 0.09	0.25 ± 0.12	0.12 ± 0.05	0.11 ± 0.03	0.11 ± 0.04
K^+^	0.78 ± 0.48	0.71 ± 0.32	0.27 ± 0.09	0.66 ± 0.31	1.05 ± 0.53
NH_4_^+^	7.02 ± 5.69	5.08 ± 3.00	2.54 ± 1.92	7.36 ± 6.06	9.33 ± 6.11
F^−^	0.22 ± 0.11	0.06 ± 0.06	0.24 ± 0.04	0.28 ± 0.05	0.26 ± 0.09
NO_3_^−^	10.86 ± 8.72	12.08 ± 9.53	2.87 ± 1.88	12.07 ± 9.32	12.32 ± 7.87
SO_4_^2−^	6.63 ± 4.07	6.46 ± 3.84	3.24 ± 2.04	6.59 ± 4.07	7.89 ± 4.01
SIA/WSIIs (%)	78 ± 11	78 ± 10	69 ± 11	78 ± 15	81 ± 7
SIA/PM_2.5_ (%)	35 ± 14	45 ± 15	25 ± 13	29 ± 14	38 ± 12
WSIIs/PM_2.5_ (%)	44 ± 16	57 ± 16	35 ± 15	35 ± 14	47 ± 14

**Table 2:** Mass concentration of WSIIs and PM_2.5_ in Botou and other cities (μg/m^3^).

Sites	Sampling Time	PM_2.5_	F^−^	Cl^−^	NO_3_^−^	SO_4_^2−^	Na^+^	NH_4_^+^	K^+^	Mg^2+^	Ca^2+^
Botou	2018.3–2019.2	79.15 ± 48.44	0.22 ± 0.11	2.51 ± 1.79	10.86 ± 8.72	6.63 ± 4.07	0.48 ± 0.17	7.02 ± 5.69	0.78 ± 0.48	0.14 ± 0.09	1.04 ± 0.82
Hefei [36]	2012.9–2013.8	86.29	/	1.21	15.14	15.56	0.48	7.82	0.96	0.30	5.24
Handan [37]	2013	139.4	/	4.4	20.6	25.2	0.7	13.0	1.8	0.1	1.0
Handan [37]	2014	116.0	/	4.5	16.7	17.8	0.6	14.4	2.0	0.2	1.0
Xi’an [38]	2018.3–2018.10	134.9	0.10	1.5	12.1	7.6	0.75	4.5	0.89	0.26	4.7
Taiyuan [39]	2017.8–2016.5	109.6	/	3.4	13.1	19.1	0.5	12.7	1.3	0.8	2.6
Urumqi [40]	2017.9–2018.8	158.85	0.52	0.37	13.46	13.58	1.93	10.88	0.25	0.22	1.93

## Data Availability

The data presented in this study are available on request from the corresponding author.

## Abbreviations

WSIIs	water-soluble inorganic ions
PMF	Positive Matrix Factorization
BTH	Beijing–Tianjin–Hebei
SOR	Sulfur Oxidation Ratio
NOR	Nitrogen Oxidation Ratio
SIA	secondary inorganic aerosols
